# Molecular events in the jaw vascular unit: A traditional review of the mechanisms involved in inflammatory jaw bone diseases

**DOI:** 10.7555/JBR.36.20220266

**Published:** 2023-04-28

**Authors:** Ruyu Wang, Haoran Wang, Junyu Mu, Hua Yuan, Yongchu Pang, Yuli Wang, Yifei Du, Feng Han

**Affiliations:** 1 Department of Oral and Maxillofacial Surgery, the Affiliated Stomatological Hospital of Nanjing Medical University, Nanjing, Jiangsu 210029, China; 2 Jiangsu Province Key Laboratory of Oral Diseases, Nanjing Medical University, Nanjing, Jiangsu 210029, China; 3 Jiangsu Province Engineering Research Center of Stomatological Translational Medicine, Nanjing, Jiangsu 210029, China; 4 International Joint Laboratory for Drug Target of Critical Illnesses, Key Laboratory of Cardiovascular and Cerebrovascular Medicine, School of Pharmacy, Nanjing Medical University, Nanjing, Jiangsu 211166, China; 5 Department of Orthodontics, the Affiliated Stomatological Hospital of Nanjing Medical University, Nanjing, Jiangsu 210029, China

**Keywords:** inflammatory diseases, osteogenesis-related cells, immune cells, blood vessels, lymphatic vessels, jaw vascular unit

## Abstract

Inflammatory jaw bone diseases are common in stomatology, including periodontitis, peri-implantitis, medication-related osteonecrosis of the jaw, radiation osteomyelitis of the jaw, age-related osteoporosis, and other specific infections. These diseases may lead to tooth loss and maxillofacial deformities, severely affecting patients' quality of life. Over the years, the reconstruction of jaw bone deficiency caused by inflammatory diseases has emerged as a medical and socioeconomic challenge. Therefore, exploring the pathogenesis of inflammatory diseases associated with jaw bones is crucial for improving prognosis and developing new targeted therapies. Accumulating evidence indicates that the integrated bone formation and dysfunction arise from complex interactions among a network of multiple cell types, including osteoblast-associated cells, immune cells, blood vessels, and lymphatic vessels. However, the role of these different cells in the inflammatory process and the 'rules' with which they interact are still not fully understood. Although many investigations have focused on specific pathological processes and molecular events in inflammatory jaw diseases, few articles offer a perspective of integration. Here, we review the changes and mechanisms of various cell types in inflammatory jaw diseases, with the hope of providing insights to drive future research in this field.

## Introduction

Inflammatory lesions are the most common pathologic condition in jaw diseases, including periodontitis, peri-implant inflammation, medication-related osteonecrosis of the jaw (MRONJ), and osteoradionecrosis. Periodontitis is an oral disease with an overall prevalence of 45%–50% ^[ 1] ^, causing tooth loss and the resorption of alveolar bone to seriously affect patients' oral functions. Although plaque removal and reduction of inflammatory factors are the main treatments for periodontitis, an irreversible loss of attachment and alveolar bone still occurs in most cases ^[ 2] ^. As economic conditions improve, more people are choosing dental implants to repair dentition defects. The formation of bone binding is the criterion of a successful implantation; however, peri-implant inflammation, characterized by inflammation of the mucous membrane around the implant and a progressive loss of supporting bone, hinders bone binding and often leads to implant failure ^[ 3] ^.

MRONJ is a severe adverse reaction associated with antiresorptive, antiangiogenic, and targeted therapies, such as bisphosphonates ^[ 4] ^, denosumab ^[ 5] ^, sunitinib ^[ 6] ^, and everolimus ^[ 7] ^. Bisphosphonates, generally prescribed to patients with metastatic cancer or multiple myeloma, are considered one of the main causes of osteonecrosis. Conservative treatments ( *e.g.*, debridement, curettage, and isolation resection) or more radical treatments, such as borderline/segmentectomy, are often used for patients with MRONJ. However, these surgical methods are associated with some postoperative complications, such as bleeding, pain, infection, and orofacial deformities ^[ 8] ^. Radiotherapies for head and neck malignancies may also cause jaw injury and result in pain, swelling, sequestrum with persistent suppuration, fracture, *etc*. Tooth extraction after radiotherapy is recognized as a key risk factor in developing osteoradionecrosis ^[ 9– 10] ^.

The treatment of inflammatory diseases of the jaw bone is limited to removing the lesions, and such an approach cannot achieve the tissue repair. Therefore, a deeper investigation of their pathological mechanisms that are still not fully understood has become a main topic of interest among scientists. The current review has focused on the significant parts of the jaw bone, their cellular signal transduction, and intercellular communication in inflammatory diseases of the jaw in an overall and systematic way, hoping to better understand the mechanisms and provide potential insights into developing new therapeutic drugs.

## Jaw vascular unit

**Figure 1:**
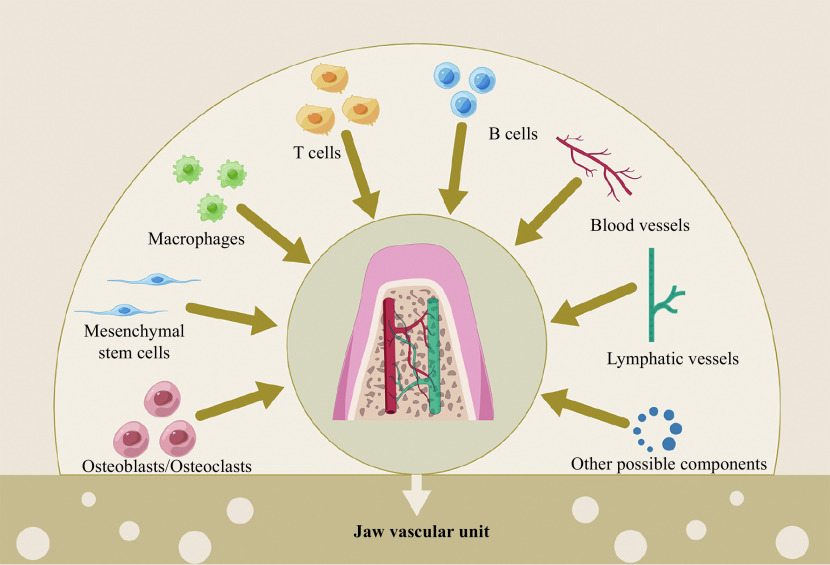
A schematic working model for the jaw vascular unit.

In the jaw bone, osteogenesis-related cells regulate bone formation ^[ 11] ^, immune cells fight infection ^[ 12] ^, and blood and lymphatic vessels maintain internal environmental homeostasis ^[ 13– 14] ^. Although each action appears to have a specific role, there are clear interactions between cells and correlated factors. For example, one study has found that immune cells can regulate osteoblasts/osteoclasts balance to influence bone repair ^[ 15] ^. Additionally, lymphatic vessels promote the remission of inflammatory responses by recruiting immune cells that secrete corresponding active factors to promote lymphatic vessel formation ^[ 14] ^. Therefore, the correlation among osteogenesis-related cells, immune cells, blood vessels, and lymphatic vessels has a crucial role in the disease development. Herein, we first propose the concept of the jaw vascular unit (JVU) that contains osteogenesis-related cells, immune cells, blood vessels, and lymphatic vessels. Such a JVU may be helpful for better understanding the pathogenesis of inflammatory diseases of the jaw bone and for discovering new therapeutic regimens ( ***Fig. 1***).

## The importance of the JVU components in bone formation and disease

### Osteogenesis-related cells in the JVU

#### Osteoblasts

The bone repair process results from a regulated balance between two bone cell populations, *i.e.*, the dynamic balancing process of osteoblasts and osteoclasts. Osteoblasts are mainly found in the bone marrow and derived from mesenchymal stem cells (MSCs). Their main functions as the cells involved in bone formation include the secretion, synthesis, and mineralization of bone matrices. Studies have found that osteoblasts have a positive feedback effect on MSCs. For example, the co-culture of mature osteoblasts with MSCs could lead to the osteogenic differentiation of MSCs ^[ 16] ^. Osteoblasts are also sources of bone protective protein that bind to the receptor activator of nuclear factor kappa-B ligand (RANKL) and prevent its binding to RANK, a process that in turn inhibits the osteoclast-mediated bone resorption ^[ 17] ^. Additionally, the tumor necrosis factor-α (TNF-α) converting enzyme, a kind of nuclear transcription factor-κB (NF-κB) ligand that stimulates RANKL expression in osteoblasts, promotes bone formation and increases inflammatory bone resorption ^[ 18] ^. Conversely, an increased SEMA3A expression in osteoblast cells may reverse bone resorption, which is strictly regulated by the NRP1-RANKL axis ^[ 19] ^.

#### Osteoclasts

Osteoclasts, derived from the monocyte-macrophage system, are the main cells involved in bone resorption ^[ 11] ^. Macrophage colony-stimulating factor (M-CSF) and RANKL are essential for osteoclast formation ^[ 20] ^. Briefly, M-CSF binds to its receptor, c-Fms, to send signals that induce RANKL expression and contribute to the proliferation and differentiation of osteoclast precursor cells ^[ 21] ^. MERproto-oncogene,tyrosinekinase (MERTK) and TYRO3 protein tyrosine kinase (TYRO3), two key receptors in the TAM family of tyrosine kinases, regulate the differentiation and migration of osteoclasts ^[ 22] ^. Genetic analysis of osteoclasts also indicates that decreased expression of Gα13 activates AKT/GSK3β/NFATc1 signaling and contributes to a dramatic increase in the number and activity of osteoclasts ^[ 23] ^.

#### Osteocytes

Osteocytes are dendritic-shaped cells embedded in the bone matrix and regulate bone formation and resorption. One study has found that osteocytes play an important role in the bone defect reparation and periodontitis. They may increase bone formation by synthesizing bone matrix that is subsequently mineralized ^[ 24] ^. Nevertheless, the accumulation of oxidative stress biomarkers, such as reactive oxygen species, leads to osteocyte apoptosis and bone loss ^[ 25 **–** 26] ^.

#### Mesenchymal stem cells

Stem cells are a class of cells with an unlimited or immortal self-renewal capacity that can produce at least one type of highly differentiated progeny. Stem cell-based therapies are of an emerging but rapidly expanding field that has seeped into the periodontal field, as it potentially overcomes the limitations of traditional regenerative procedures ^[ 27] ^. MSCs can differentiate into various tissues and are easily isolated from bone marrow or adipose tissue, which can be easily expanded *in vitro* to sufficient numbers for clinical application ^[ 28] ^. MSCs are pluripotent cells regulated by different molecular factors that control their direction of differentiation ^[ 29] ^. Differentiation of the osteogenic spectrum is mainly affected by the Wnt/β-catenin signaling pathway and the bone morphogenetic protein (BMP) formation pathway.

Ling *et al* have discovered that the Wnt/β-catenin pathway regulates MSCs osteogenesis in inflammatory diseases ^[ 30] ^. Activation of the Wnt pathway promotes the osteogenic differentiation of periodontal stem cells, while its inhibition hinders this phenomenon ^[ 31] ^. Moreover, osteogenesis of the bone marrow-derived mesenchymal stem cells (BMSCs) may be promoted *in vitro* by activating the WNT/β-catenin linked protein signaling pathway. Also, a conditioned medium for MSCs (MSC-CM) can promote periodontal tissue regeneration. For example, the topical application of MSC-CM to rats with periodontitis revealed new bone and new periodontal ligaments after four weeks ^[ 32] ^. It was also found that the regenerative potential of MSC-CM for periodontal tissues was mainly synergistically mediated by the insulin-like growth factor-1 (IGF-1), vascular endothelial growth factor (VEGF), and transforming growth factor-β1 (TGF-β1) ^[ 33– 34] ^. Exosomes from MSCs also promote the proliferation and osteogenesis of osteoblasts, and relieve the migration and proliferation of periodontal ligament cells through the AKT and ERK signaling, which provide a potential strategy for treating periodontitis ^[ 35– 36] ^.

#### Interrelated events in osteogenesis-related cells

In inflammatory diseases like rheumatoid arthritis, periodontitis and osteoporosis, the homeostasis is lost and bone resorption occurs. The RANKL expression represents the osteoclast-osteoblast homeostasis. In the periodontitis cell model, this ratio is reduced ^[ 37] ^. Furthermore, RUNX2 is essential for osteoblast differentiation and chondrocyte maturation ^[ 38] ^. RUNX2 is weakly expressed in immature mesenchymal cells during osteoblast differentiation, and its expression is upregulated in pre-osteoblast and reaches its maximum level in immature osteoblasts ^[ 39] ^. RUNX1 is expressed at different stages of the chondrocyte and osteoblast differentiation, and the *Runx1* chondrocyte-specific knockout mice exhibited an impaired chondrogenesis and a decreased bone density ^[ 40] ^. Osteocalcin (OCN) is a marker of osteoblast differentiation and maturation, and alkaline phosphatase (ALP) is the main component of osteoblast extracellular matrix. The conditional knockout of the *PDK-1* gene in osteoblasts significantly inhibits the expression of osteogenesis-related proteins, including OCN and ALP ^[ 41] ^.

Studies have found molecular events that integrate growth control, cell fate programming, and positional information yield the exquisite replacement of the amputated limb ^[ 42] ^. For example, Geurtzen *et al* confirmed that osteoblast differentiation helped fins and skull defect bone regeneration in zebrafish ^[ 43] ^. In 2020, Mishra *et al* ^[ 44]^ discovered that the NF-κB signal suppression resulted in more undifferentiated RUNX2 positive cell recruitment to the regeneration of cells, promoting osteoblast differentiation and bone regeneration in zebrafish. It has also been found that a β3 integrin-FAK-ERK1/2-RUNX2 pathway can be activated by DEL-1, leading to osteoblast formation and new bone formation ^[ 45] ^.

miRNA plays a major role in regulating the progression of bone formation. Studies have shown that the overexpression of miR-130a increases the osteogenic differentiation of BMSCs, whereas miR-130a inhibition reduces osteogenic differentiation and promotes lipid droplet formation ^[ 46] ^. miR-155 from the extracellular vesicles of M1 macrophages inhibits MSC osteogenesis and osteogenic marker expression ^[ 47] ^. However, miR-146a can alleviate osteoblast loss in osteomyelitis caused by staphylococcus aureus and then regulate bone remodeling and inhibit the production of inflammatory cytokines ^[ 48] ^. A study showed that glucocorticoid exerted its function by binding to the glucocorticoid receptor; the physiological concentration of glucocorticoid stimulated osteoblast proliferation and promoted the osteogenic differentiation of MSCs through the Wnt, TGFβ/BMP superfamily and Notch signaling pathways as well as transcription factors, post-transcriptional regulators, and other regulators that control the osteogenic differentiation of MSCs mediated by glucocorticoid receptor ^[ 49] ^. In recent years, therapeutic potential of resveratrol has been discovered, mostly for the prevention and treatment of bone loss diseases ^[ 50] ^. One study has shown that resveratrol can induce gingival stem cell autophagy and promote osteogenic differentiation of gingival stem cells by activating the AMPK-BECLIN1 pathway, confirming that the regulation of autophagy contributes to the differentiation of gingival stem cells into osteoblasts and can be transformed into regenerative cell therapy for jaw defects ^[ 51] ^. Another study has also shown that miR-135-5p promotes osteogenic differentiation and calcification and increases the levels of calcification and osteogenic markers, including RUNX2, OSX, OPN, and OCN ^[ 52] ^. In addition, as an energy supplier to cells, glycogen is a signaling molecule that activates cells. By activating AKT/GSK‐3β, glycogen stimulates preosteoblasts, promoting osteoblast differentiation and bone formation ^[ 53] ^.

### Immune cells in the JVU

#### Macrophages

Macrophages contribute to tissue homeostasis and defense functions ^[ 54] ^. Yet, excessive macrophage aggregation can lead to tissue damage. Also, bacteria and their products can activate macrophages to produce pro-inflammatory factors that can cause inflammation and initiate immune responses, leading to soft tissue inflammation and jaw bone tissue resorption ^[ 55] ^. Macrophage polarization can be divided into two categories according to their function: the M1 type that promotes inflammation, and the M2 type that promotes tissue repair and vascularization. It has been demonstrated that C-C motif chemokine ligand 2 (CCL2) promotes M1-type polarization to M2-type, further suppressing inflammatory bone loss ^[ 56] ^. In addition, cellular experiments using a mouse macrophage cell line, RAW264.7, induced with CCL2 for 48 h and monitored by flow cytometry monitoring, showed an average increase of 4.177% in the CD206 ^+^/CD86 ^−^ cell population, confirming the positive effect of M2-type macrophages on bone tissue repair in periodontitis ^[ 56] ^. Other studies have found that in infectious osteomyelitis, bacterial virulence factors, such as lipopolysaccharide, induce macrophages to polarize toward M1-type, and exert bactericidal and phagocytic effects, but also lead to tissue damage and osteoclast activation, triggering bone resorption; in the anti-infective phase, the presence of M2-type macrophages promotes tissue repair ^[ 57– 58] ^. M1-type macrophages contribute to the osteoclast activation by stimulating cytokines, such as PGE2, IL-1β, TNF-α, IL-6, and IL-12, aggravating periodontitis processes and bone resorption ^[ 59– 60] ^. Analysis of the MRONJ mouse model also shows the increased M1-type macrophages and the decreased M2-type macrophages in the mucoperiosteum adjacent ^[ 61] ^.

The ratio of M1 to M2 types can reflect the health of jawbone tissue to some extent, *i.e.*, an increased M1/M2 ratio can promote jawbone resorption, and *vice versa* ^[ 62] ^. The PI3K/AKT pathway, as one of the main signaling pathways regulating macrophages, has received much attention in regulating M2 polarization, and has been shown to mediate M2 polarization ^[ 63] ^. It has been demonstrated that the deletion of GAB1 and GAB2 in the GAB family of signaling anchor proteins hinders M2 polarization in macrophages, whereas IL-4 can participate in M2 polarization in macrophages by activating GAB1, thus inducing the AKT signaling pathway ^[ 64] ^.

M2 macrophages are important for promoting osteoblast differentiation and osteogenesis ^[ 65] ^. The amount of BMP-2 secreted by M2 macrophages is higher than that by M0 or M1 macrophages, which contributes to the osteoblastic ability induced by M2 macrophages and the differentiation of MSCs ^[ 66] ^. Reparative M2-like macrophages can promote tissue repair and prevent bone loss ^[ 67] ^. Moreover, M2-Exos can promote the osteogenic differentiation of bone marrow mesenchymal stem cells but inhibit osteoclast formation, jaw resorption and collection due to intercellular communication *via* exosomes ^[ 68] ^. In addition, the transport of magnesium ions contributes to the infiltration and activation of CD68 macrophages in bone defects. The nuclear translocation of M7CK *via* the TRPM7 channel leads to the polarization of macrophages into the reparative M2 type, which promotes the recruitment and osteogenic differentiation of MSCs, thus achieving the role of repairing bone defects ^[ 69] ^.

#### CD4 T cells

CD4 helper T cells, one of the key factors in the immune system, play a regulatory role in the defense of human immunity ^[ 70] ^. It has been demonstrated that cellular immunity is a major component in our inflammatory immune responses ^[ 71] ^. Multiple signaling pathways influence the differentiation of CD4 T cells. For example, when stimulated by bacteria and viruses, immature CD4 T cells can differentiate into multiple subtypes, including Th1, Th2, Th17, and regulatory T (Treg) cells. Th1 is involved in the developmental process of periodontitis, and osteoprotegerin ligands can be expressed in Th1 and then stimulate osteoclast differentiation ^[ 72] ^; the activated Th2 can secrete various cytokines, which in turn promote B cell activation and proliferation of B cells to mediate humoral immunity ^[ 73] ^; Th17 is an osteoclast T cell that exacerbates inflammation response by secreting the inflammatory factor IL-17. Treg can secrete IL-10 that inhibits the secretion of inflammatory factors and has a certain tissue repair effect, both of which are important for maintaining the internal immune environment ^[ 74– 75] ^. Senescent CD4 ^+^ CD28 ^−^ T lymphocytes have been investigated as a common feature of chronic bone resorption diseases, such as rheumatoid arthritis, osteoporosis, and osteomyelitis ^[ 76] ^, and these lymphocytes may also play a role in the pathogenesis of jaw inflammatory diseases, such as periodontitis or MRONJ.

In mice with periodontitis, the increased Th17-related genes ( *i.e.*, *Il6*, *Il17a*, and *Rorγt*), and Treg-related genes ( *i.e.*, *Foxp3*, *Il2*, *Il10*, *Ctla4*, *Cd25*, and *Gitr*) were detected in cervical lymph nodes and spleen ^[ 77] ^. An imbalance of the Th17/Treg ratio in the body causes an increased inflammatory response. A clinical study examined the blood and periodontal tissues from healthy individuals and patients with periodontitis by ELISA, and the results showed an elevated IL-17 level and a significantly lower IL-10 level in patients with periodontitis, confirming the role of Th17/Treg balance in the progression of periodontitis disease ^[ 78] ^.

Furthermore, the Th17-derived extracellular vesicles can induce osteoclast progenitors to differentiate into osteoclasts, and miR-132 in extracellular vesicles of Th17 can induce osteoclast progenitors to differentiate into osteoclasts ^[ 79] ^. Also, the probiotic nutritional supplement can prevent pathological bone loss. Previous studies have demonstrated that *Lactobacillus rhamnose*-GG can increase Treg cells to stimulate CD8 T cells to secrete WNT ligand WNT10b, promoting bone repair ^[ 80] ^.

#### B cells

B cells produce and release antibodies against specific antigens, and are important components of the humoral immune responses. The production of B cells begins in bone marrow, where stem cells can give rise to lymphocytes. Inflammatory infiltration of B cells (especially plasma cells) is the hallmark of periodontitis. B cells in periodontitis mainly secrete IgG specific to periodontal pathogens. Memory B cells are located under the connective tissue of the healthy gingival junction epithelium and maintain periodontal homeostasis ^[ 81] ^. The main B cell subsets, include B1 cells, B2 cells, and regulatory B (Breg) cells. B1 cells are mainly found in fetuses and neonates and function like plasma cells; B2 cells are mainly derived from bone marrow and can further differentiate into follicular B cells and marginal zone B cells; Breg cells produce the anti-inflammatory factor IL-10 to suppress the immune response ^[ 82] ^.

It has recently been shown that Breg cells inhibit periodontal inflammation and bone resorption in experimental periodontitis mice ^[ 83– 84] ^. The periodontitis modeling using B-cell-deficient CD19Cre mice showed that B-cell deficiency exacerbated periodontal bone resorption, compared with the wild-type mice, and the elevated levels of Th1 cytokines and RANKl were found in the gums of CD19Cre mice, confirming that B-cell deficiency activates Th1 and osteoclasts, which exacerbates the progression of periodontitis ^[ 85] ^. Furthermore, some other studies have shown that IL-10 produced by B cells can alleviate alveolar bone resorption and regulate the local CD4 ^+^ T cell population, suggesting that B cells have some potential for regulating periodontitis ^[ 86] ^. Han *et al* ^[ 87]^ reported a higher degree of alveolar bone resorption in mice injected with Breg activator-TLR than in mice with periodontitis alone. Also, it was found that IL-1β, IFN-γ, and IL-17 were lower in the gingiva of mice injected with Breg cells than in those without Breg cells, confirming that Breg cells can inhibit not only the progression of the periodontitis but also regulate the T cell population, *i.e.*, the expression levels of Th1/Th17.

The efficacy of IL-10 cytokine also leads to various inflammatory bone loss disorders. IL-10 is a signature cytokine of Breg cells and an effective regulator of the immune response, further contributing to the maintenance of bone health by inhibiting osteoclast-mediated bone resorption ^[ 88] ^.

### Blood vessels in the JVU

The vascular endothelium is composed of endothelial cells that form the inner wall of arterial, vein, and capillary endothelial cells, which are in direct contact with blood components and are the mainstay of the constituent vessels ^[ 89] ^. Angiogenesis, the formation of new blood vessels, accelerates the process of wound healing ^[ 90] ^. In treating inflammatory jaw bone diseases, the most difficult problem is to solve jaw defects, and the study of angiogenesis for bone repair is an emerging research direction. One study has found that blood vessels promote tissue development or bone regeneration by perfusing the healing zone during physiological or bone regeneration to facilitate adequate nutrients, growth factors, and oxygen for tissue regeneration and bone repair ^[ 13] ^. H-type vessels, a specific capillary subtype coupling angiogenesis and osteogenesis in rodents and humans, have been fully investigated in the treatment of bone absorption diseases ^[ 91] ^.

VEGF and BMP are important regulators of angiogenesis, they have a key role in vascular development and tissue regeneration ^[ 92] ^. VEGF can promote endothelial cell migration and proliferation and indirectly stimulate osteogenesis and angiogenesis through paracrine effects ^[ 93] ^. Several studies have shown that the VEGF protein can increase bone formation in mice ^[ 94] ^. Moreover, osteogenesis and angiogenesis are interrelated; the use of an active substance, *i.e.*, fucoidan, can promote the osteogenic differentiation of BMSCs, while the use of a conditioned medium for the induction of osteogenesis of BMSCs can induce the formation of blood vessels in umbilical vein endothelial cells ^[ 95] ^.

Angiogenesis is essential for bone tissue regeneration. Besides osteoblasts, osteoclasts and chondrocytes, endothelial cells greatly affect bone tissue regeneration. Without endothelial cells, bone tissue regeneration tends to affect bone morphology, length, and quality ^[ 96] ^. Studies have demonstrated that Notch signaling plays an important role in regulating angiogenesis, for example, blocking Notch signaling in a mouse model resulted in an abnormal vascular morphology and reduced osteogenesis ^[ 97] ^. Moreover, the zinc-finger E-box-binding homeobox 1 (ZEB1), a transcription factor that regulates cell features, can trigger epithelial-mesenchymal transformation and is critical for developing a range of pathological states ^[ 98– 99] ^. A study has shown that endothelial cell-specific loss of ZEB1 reduces vascularization and osteogenesis in aging and osteoporotic mice as well as in patients with osteoporosis, reducing the H-type blood vessel formation ^[ 100] ^.

miRNAs in exosomes secreted by MSCs, such as miR-126, miR-130a, and miR-132, have been found to play important roles in regulating vascular endothelial cell proliferation and angiogenesis ^[ 101] ^. For example, miR-126 in exosomes from endothelial progenitor cells promotes endothelial cell proliferation, migration, and angiogenesis ^[ 102] ^. Extracellular vesicles of dental pulp stem cells have been found to transfer miR-378a into endothelial cells, thereby activating the Hedgehog/Gli1 signaling pathway to promote endothelial cell proliferation, migration, and angiogenesis ^[ 103]^

### Lymphatic vessels in the JVU

Lymphatic vessels, which are widely distributed in all body tissues, are an essential part of the circulatory system and have an important role in human immunity. Alterations in lymphatic vessels can lead to the development of many diseases. The lymphatic vascular system maintains tissue fluid homeostasis and participates in lipid transport in normal physiological conditions. Additionally, it is closely correlated with tumor metastasis, wound healing, and inflammatory responses ^[ 104] ^. The main difference between lymphatic vessels and blood vessels is that blood vessels are composed of blood vascular endothelial cells, which are tightly connected and have a continuous basement membrane, whereas lymphatic vessels are composed of lymphatic endothelial cells, which are not directly connected and have a discontinuous basement membrane ^[ 105] ^. Lymphatic circulation, like blood circulation, is crucial for maintaining the homeostasis of the internal environment and for the operation of normal physiological processes in the body.

Lymphatic vessel renewal is correlated with inflammation and tissue damage. Its role is to enhance lymphatic return, immune cell transit, and associated antigen clearance. A previous study showed increased lymphatic and vascular vessels in the periodontal tissues of mice with the periodontitis model, compared with normal mice. Additionally, lymphatic vessels remained dilated and recruited immune cells after the formation of chronic periodontitis, which may be correlated with tissue repair ^[ 14] ^. Monocytes/macrophages contribute to the lymphatic vessel formation during inflammation and reduce tissue edema in wound healing. In chronic obstructive pulmonary disease, bronchitis, myocardial infarction, and hepatitis, multiple immune cells are activated in inflammatory states, expressing lymphangiogenesis-related factors (VEGF-C and -D) that in some circumstances can alleviate the inflammatory response associated with the diseases, suggesting that lymphangiogenesis may be a promising disease treatment prospect ^[ 106– 108] ^.

The secretion of CXCL12 by lymphatic endothelial cells is critical for hematopoietic and bone regeneration. CXCL12 triggers the expansion of mature Myh11 ^+^CXCR4 ^+^ pericytes that differentiate into osteoblasts and contribute to bone formation ^[ 109] ^. VEGF-C and its homologous receptor VEGFR-3 are expressed in lymphatic smooth muscle cells and are major regulatory factors of lymphatic angiogenesis ^[ 110] ^. Macrophages are the main source of VEGF-C, and the activation of macrophages and T cells interaction can enhance the expression of VEGF-C ^[ 111]^ controlling the lymphatic generation and immune cell migration. Studies have shown that lymphangiogenesis (mainly involving lymphatic capillary vessels) is obvious during acute inflammation. In chronic inflammation progression, the number of lymphatic vessels is significantly reduced, and the majority of lymphatic vessels are mature lymphatic vessels. In synovial tissue samples of patients with inflammatory joints, the number of lymphatic vessels and the level of VEGFR-3 expression are increased. Shi *et al* investigated the changes of lymphatic vessels in knee joints of normal and osteoarthritic mice ^[ 112]^ and found that the number of mature vessels in the joint of mice with severe osteoarthritis was significantly reduced and that the tissue space was damaged. They also provided valuable insights into the treatment of jaw inflammatory diseases.

## Cell-cell signaling in the JVU

Bone formation is a multi-step complex process that depends on interactions among osteogenesis-related cells, immune cells, blood vessels, and lymphatic vessels. During pathological process of inflammatory jaw-bone diseases, the main components of the JVU include cooperation and response mechanism. The analysis of periodontally involved tissues from patients with periodontitis without systemic diseases revealed a significantly higher abundance of M1-type macrophages and a lower abundance of M2-type macrophages in the tissues ^[ 12] ^. The lower abundance of M2 macrophages reduced BMP-2 secreting and further inhibited osteoblast differentiation of MSCs ^[ 66] ^. In addition, the B-cell deficiency contributed to the upregulating of Th1 cytokines and RANKL, which directly activated osteoclasts and induced periodontitis ^[ 85] ^. In MRONJ, the exosomes from abnormal macrophages could be taken up by endothelial cells, thereby preventing the formation of type H blood vessels. Moreover, abnormal macrophages in MRONJ slowed an osteoclastic activity and impaired the removement of necrotic bone tissue ^[ 113] ^.

**Figure 2:**
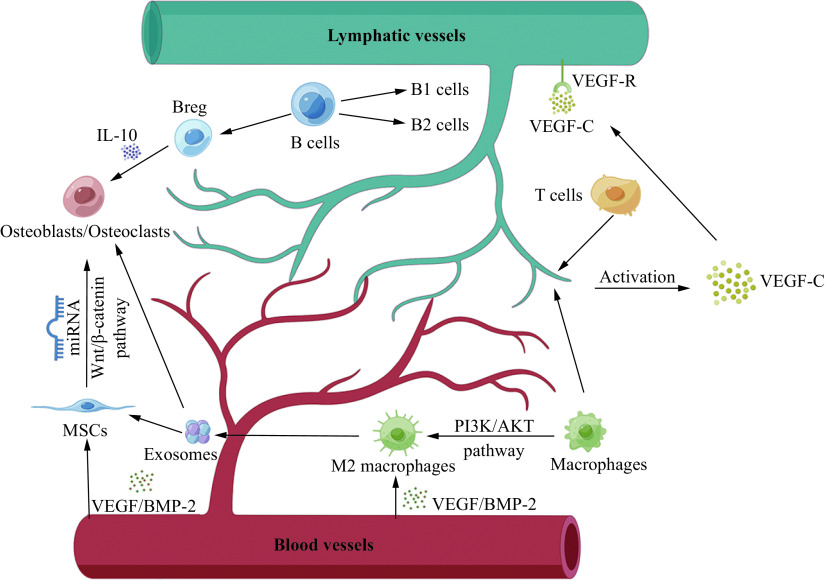
A schematic working model for the important components and cell-cell signaling in the jaw vascular unit.

The inflammatory microenvironment also causes lymphatic vessel dysfunction, which promotes osteoclast formation and contributes to alveolar bone loss. One study found that VEGF-C, the major growth factor that induces lymphangiogenesis, enhanced local lymphatic vessel formation and reversed alveolar bone loss ^[ 114] ^. Another study found that VEGF-C also increased the expression of osteogenic markers and promoted the mineralization of MSCs, confirming the potential influence of lymphangiogenesis on osteogenesis ^[ 115] ^. From the perspective of systems biology, we may simulate the complex clinical pathological processes to resolve the underlying mechanisms quite precisely by utilizing the concept of the JVU ( ***Fig. 2***).

## Conclusions and perspectives

Osteoblasts are the main functional cells in bone formation, responsible for the secretion, synthesis, and mineralization of bone matrices. Osteoclasts are derived from the monocyte-macrophage system and are the main cells involved in bone resorption. Osteocytes are dendritic-shaped cells embedded in the bone matrix and express several pro-inflammatory cytokines linked to the osteoclast-mediated bone resorption. MSCs can differentiate into various tissues and are easily isolated from bone marrow or adipose tissue and can also be easily expanded *in vitro* to sufficient numbers for clinical osteogenesis applications. Pro-inflammatory and inflammatory factors secreted by different types of macrophages can cause inflammation or anti-inflammation response, leading to the resorption and formation of the jaw bone. Abnormal CD4 helper T cells cause the inflammatory response in the jaw bone. Inflammatory infiltration of B cells is the hallmark of various jaw inflammatory diseases. Angiogenesis, the formation of new blood vessels, accelerates the process of functional jaw reconstruction. The lymphatic vascular system can maintain tissue fluid homeostasis and participate in lipid transport, which is closely correlated with inflammatory responses and the jaw bone formation. Osteogenesis-related cells, immune cells, blood vessels, and lymphatic vessels are the main components of the JVU, which may interact with each other. For example, immune cells adjust the balance of osteoblasts and osteoclasts by promoting bone formation ^[ 116] ^; blood vessels promote bone formation by nourishing surrounding tissues and secreting VEGF and BMP ^[ 92] ^; and lymphatic vessels promote the remission of inflammation by recruiting immune cells that secrete active factors to promote the formation of lymphatic vessels ^[ 117] ^. If any of these processes is altered, bone formation can be affected. The impairment of one or more parts in the JVU may evaluate the pathological process of jaw bone inflammatory diseases. Therefore, a better understanding of the interaction networks in the JVU is useful for finding new treatment targets and formulating new therapies for treating jaw bone inflammatory diseases.
